# The *ybcF* Gene of *Escherichia coli* Encodes a Local Orphan Enzyme, Catabolic Carbamate Kinase

**DOI:** 10.4014/jmb.2210.10037

**Published:** 2022-11-07

**Authors:** Nam Yeun Kim, Ok Bin Kim

**Affiliations:** Department of Life Science, Division of EcoScience, Ewha Womans University, Seoul 03760, Republic of Korea

**Keywords:** *ybcF*, catabolic carbamate kinase, carbamoyl phosphate, allantoin

## Abstract

*Escherichia coli* can use allantoin as its sole nitrogen source under anaerobic conditions. The ureidoglycolate produced by double release of ammonia from allantoin can flow into either the glyoxylate shunt or further catabolic transcarbamoylation. Although the former pathway is well studied, the genes of the latter (catabolic) pathway are not known. In the catabolic pathway, ureidoglycolate is finally converted to carbamoyl phosphate (CP) and oxamate, and then CP is dephosphorylated to carbamate by a catabolic carbamate kinase (CK), whereby ATP is formed. We identified the *ybcF* gene in a gene cluster containing *fdrA*-*ylbE*-*ylbF*-*ybcF* that is located downstream of the *allDCE*-operon. Reverse transcription PCR of total mRNA confirmed that the genes *fdrA*, *ylbE*, *ylbF*, and *ybcF* are co-transcribed. Deletion of *ybcF* caused only a slight increase in metabolic flow into the glyoxylate pathway, probably because CP was used to de novo synthesize pyrimidine and arginine. The activity of the catabolic CK was analyzed using purified YbcF protein. The *V*_max_ is 1.82 U/mg YbcF for CP and 1.94 U/mg YbcF for ADP, and the *K*_M_ value is 0.47 mM for CP and 0.43 mM for ADP. With these results, it was experimentally revealed that the *ybcF* gene of *E. coli* encodes catabolic CK, which completes anaerobic allantoin degradation through substrate-level phosphorylation. Therefore, we suggest renaming the *ybcF* gene as *allK*.

## Introduction

Carbamoyl phosphate (CP) is an essential metabolite involved in the biosynthesis of pyrimidine nucleotide and arginine/urea and also in the synthesis of antibiotics [1-3]. CP is composed of only three functional groups, ammonia, carbonate, and phosphate, and it is metabolically reactive with high energy. Walsh *et al*. called CP one of the eight key metabolites at the core of cell metabolism [4].

CP is produced in various pathways using several unique enzymes. The first pathway is irreversible formation of CP by carbamoyl phosphate synthetase (CPS) from ammonia (or glutamine), bicarbonate, and ATP, observed in most organisms. In the urea cycle of mammalian mitochondria, CP is formed from inorganic ammonium and bicarbonate by CPS catalysis, which consumes 2 ATP (reaction 1). This CPS using inorganic ammonia as the nitrogen donor is called CPS I:

$${\text{NH}}_{\text{4}}^{\text{+}}{\text{+HCO}}_{\text{3}}^{\text{-}}\text{+2ATP}\to {\text{CP+2ADP+PO}}_{\text{3}}^{\text{-}}{\text{+2H}}^{\text{+}}\text{(CPS I,E.C. 6.3.4.16)}$$ (1)

The biosynthesis of arginine and pyrimidine uses CP produced by CPS to catalyze the transfer of ammonia from glutamine to bicarbonate and also requires 2 ATP (reaction 2). This type of CPS using glutamine as the nitrogen donor is called CPS II:

$${}_{\text{L}}{\text{-Gln+HCO}}_{\text{3}}^{\text{-}}{\text{+2ATP+H}}_{\text{2}}\text{O}\to {\text{CP+}}_{\text{L}}{\text{-Glu+2ADP+PO}}_{\text{3}}^{\text{-}}{\text{+2H}}^{\text{+}}\text{(CPS II, EC 6.3.5.5)}$$ (2)

The best studied example of CPS II is in *E. coli*, where it is encoded by *carAB* (b0032-3) and forms a heterodimer composed of a CarA subunit (40 kDa, glutaminase) and a CarB subunit (120 kDa, synthase) [5].

The second path of CP formation is catalyzed by carbamate kinase-like CPS (CK-like CPS) and occurs in only a few organisms that inhabit unusual environments. For example, in some hyperthemophilic archaea such as *Pyrococcus abyssi*, *P. furiosus*, and *Thermococcus kodakarensis*, CP is produced directly from carbamate and ATP by this type of enzyme [3, 6, 7]. This form is called CK-like CPS because it functions as CPS although the enzyme is structurally similar to catabolic CK [8]. The formation of CP by CK-like CPS is an endergonic reaction under standard conditions, and it is only possible at a high concentration of NH_3_ (reactions 3 and 4), under which carbamate forms spontaneously from ammonia and bicarbonate [6]:

$$\text{Carbamate + ATP}↔\text{CP+ADP (CK-like CPS, EC 2.7.2.2.)}\Delta {\text{G}}^{0'}=\text{+8.9kJ/mol}$$ (3)

$${\text{NH}}_{\text{4}}^{\text{+}}{\text{+HCO}}_{\text{3}}^{\text{-}}↔{\text{Carbamate + H}}_{\text{2}}{\text{O (Chemical) ΔG}}^{\text{0'}}\text{=-1.6kJ/mol}$$ (4)

The third case of CP generation uses a degradation (catabolic) pathway rather than a biosynthetic (anabolic) pathway. Shi *et al*. described this pathway as a collection of catabolic transcarbamylase reactions [3]. One well-studied example is CP production by catabolic ornithine transcarbamylase (OTCase) in the arginine deiminase (ADI) pathway, which is found in *Pseudomonas aeruginosa*, *Enterococcus faecalis*, and in several bacteria in the order *Lactobacillales* [9-12]. In *Lactobacillus brevis*, arginine is anaerobically degraded by ADI into ammonia and citrulline that is further converted into ornithine and CP by OTCase [12]. These are coupled with the reaction of catabolic CK, which transfers a phosphate group to generate ATP and leave carbamate (CP + ADP → Carbamate + ATP: catabolic CK, E.C. 2.7.2.2). In this pathway, CP is formed by OTCase and used for substrate-level phosphorylation, a thermodynamically favorable pathway. Moreover, in the genome of *L. brevis*, the gene encoding CK is clustered with the genes for ADI, OTCase, and the related enzymes [12], implying that the main function of CK is related to catabolism in this strain. A similar catabolic transcarbamylase reaction produces CP by anaerobic agmatine degradation via agmatine deiminase [13, 14].

*E. coli* is another organism that uses catabolic transcarbamylase to produce CP via the oxamic transcarbamylase (OXTCase) reaction in anaerobic allantoin degradation. It has long been known that this pathway exist in *E. coli*, but the genes for OXTCase and catabolic CK have not yet been experimentally identified. The former is a global orphan, and the latter is a local orphan. Though there have been attempts to identify the protein YgeW as OXTCase, they were not successful [15]. Recently, we presented a strong candidate gene, *fdrA*, for OXTCase with evidence that *ΔfdrA* failed to convert oxalurate to CP and oxamate [16]. Near *fdrA* lies the *ybcF* gene that Smith *et al*. also suggested as a candidate for a CK [17]. If that is correct, the gene for catabolic CK of *E. coli* is clustered with allantoin pathway, unlike the CK of *L. brevis*, in which the gene is located with the ADI pathway together. In this work, we demonstrate that the *ybcF* gene of *E. coli* belongs to an *fdrA* operon and encodes the missing catabolic CK. This study is the first to experimentally validate that YbcF of *E. coli* has catabolic carbamate kinase activity.

## Materials and Methods

### Cultivation of *E. coli* on Allantoin as the Nitrogen Source

*E. coli* K-12 MG1655 (*F– λ– ilvG– rfb-50 rph-1*) and LMB111(Δ*ybcF*) were grown on glycerol (50 mM), dimethyl sulfoxide (DMSO, 50 mM), and allantoin (20 mM) in nitrogen-deficient M9 minimal medium (_ND_M9) [18] at 37°C for 48 h under anaerobic conditions with a 95% N_2_ + 5% H_2_ gas mixture.

To produce a concentrated cell suspension, *E. coli* cells grown in the above culture conditions were harvested by centrifugation for 5 min at 5,000×*g*. The cells were immediately resuspended in nitrogen-deficient 3-(*N*-morpholino) propanesulfonic acid (MOPS) buffer (no ammonium chloride added) [19] to OD_600_ = 20 and anaerobically changed with a 95% N_2_ + 5% H_2_ gas mixture. Allantoin degradation was initiated by mixing the cells with the same volume of the MOPS containing glycerol (20 mM), DMSO (20 mM), and allantoin (20 mM), and the cell suspension was incubated at 37°C and analyzed by high performance liquid chromatography (HPLC).

### RT-PCR to Validate the Operon

Using RNAprotect bacteria reagent (76506, Qiagen, Germany) and an RNeasy mini kit (74104, Qiagen), total mRNA was isolated from *E. coli* MG1655 grown anaerobically on allantoin (20 mM), glycerol (50 mM), and DMSO (50 mM) for 48 h. The mRNA was transcribed into cDNA with a ReverTra Ace qPCR RT Kit (Toyobo, Japan) using random primers. The cDNA was amplified by PCR using four forward primers (*fdrA*-RT-F [5′- ATG ATC CAC GCC TTT ATT AAA AAA GGG -3′], *ylbE*-RT-F [5′- ATG TTT ACA TCA GTG GCG CAA G -3′], *ylbF*-RT-F [5′- ATG ACG ATC ATC CAT CCT CTG -3′], and *ybcF*-RT-F [5′- ATG AAA ACA CTG GTT GTG GC -3′]) and four reverse primers (*fdrA*-RT-R [5′- TTA TTG CAA ACG TTC TAA TAA ACG AG -3′], *ylbE*-RT-R [5′-TCA ACC AAT ACC CAT GCT TTC -3′], *ylbF*-RT-R [5′- TCA TGG TTT TCC TTG TAA TAA TTG -3′], and *ybcF*-RT-R [5′- CTA CAG CGA AAT ACA GGT CC -3′]), which are located in the genes *fdrA*, *ylbE*, *ylbF*, and *ybcF* in various combinations.

### Inactivation of *ybcF*

The *ybcF* gene in the genome of *E. coli* K-12 MG1655 (F– λ– *ilvG– rfb-50 rph-1*) was deleted using the method of Datsenko and Wanner [20]. For insertional inactivation, the PCR product of the *catR* chloramphenicol resistance gene from pKD3 was used and flanked by FRT sequences. Primers *fdrA*_H1P1 [5′- CGC TGC TGG GGT TCT GGC TTG GCC AAC AAT TAT TAC AAG GAA AAC CAT GAG TGT AGG CTG GAG CTG CTT C -3′] and *fdrA*_H2P2 [5′- GAT AAG ACG CGT CAA GCG TCG CAT CAG GCA CAA ATG TCT AAT GCC TAC GAC ATA TGA ATA TCC TCC TTA G -3′] contain parts of the regions adjacent to FRT and the 5′ and 3′ regions of the amplified *ybcF*, respectively. The strain in which *ybcF* was replaced by *catR* was designated as LMB111.

### Analysis of Fermentation Products by HPLC

Culture supernatants were analyzed using an Hitachi LaChrom Elite HPLC System (Hitachi High-Tech Corp., Japan), equipped with a pump (L-2130), column oven (L-2350), autosampler (L-2200), and an Aminex HPX-87H ion-exclusion column (300 × 7.8 mm; Bio-Rad, USA). The mobile phase was 4 mM H_2_SO_4_ supplied at a constant flow rate of 0.55 ml/min. The sample was injected with 10 μl and run for 25 min. The column temperature was adjusted to 18°C for allantoin and oxamate and 27°C for oxalate and other organic acids. The quantitative determination was carried out using an L-2490 refractive index detector.

### Expression and Purification of YbcF

The *ybcF* overexpression plasmid pCA24N::*ybcF* (JW0510-AM, NBRP, NIG, Japan), which includes six histidines at the N-terminus, was transformed to MG1655 and aerobically grown in LB broth (Difco, USA) to OD_600_ of about 0.3 at 37°C. Induction was performed with 0.5 mM isopropyl β-D-1-thiogalactopyranoside (IPTG) for 2 h at 30°C. For purification of the 6X His-tagged proteins, cell pellets were suspended in lysis buffer (20 mM Tris–Cl pH 8.3, 0.5% triton X-100, 1 mg/ml lysozyme), and cell disruption was achieved by six repetitions of vortexing with acid-washed glass beads (G1145, Sigma-Aldrich) and icing for 5 min. Cell debris was removed by centrifugation (13,000 ×*g*, 4°C, 20 min), and the supernatant cell lysate was collected. The purification of YbcF was performed via affinity chromatography with Ni-NTA Spin Columns (31014, Qiagen) according to the manufacturer’s directions. Protein concentrations were determined with the Bradford method using Protein Assay Dye (5000006, Bio-Rad). The standard curve was generated using bovine serum albumin (BSA).

### Western Blotting

Elution fractions were analyzed with sodium dodecyl sulfate–polyacrylamide gel electrophoresis (SDS-PAGE) and western blotting. Samples were subjected to SDS-PAGE (Bio-Rad) using a 12% running gel. Electrophoretic transfer was performed on an Amersham Hybond P western blotting membrane (GE Health, USA) with a Trans-Blot SD Semi-Dry Transfer Cell (Bio-Rad). After blotting (15 V, 1 h 20 min), the membrane was blocked for 1 h at room temperature. Then, the membrane was incubated overnight at 4°C with 6X His-tag monoclonal primary antibody (MA1-21315, Invitrogen, USA), which was diluted 1:2000. Subsequently, horseradish peroxidase–coupled goat anti-mouse secondary antibody (G21040, Invitrogen) was diluted 1:100000 and incubated with the membrane at room temperature for 1 h. Dyne ECL Pico Plus (GBE-P200, Dynebio, Republic of Korea) was used to detect peroxidase activity. Development was performed using an ImageQuant800 (USA).

### Carbamate Kinase Assay

The carbamate kinase activity assay was measured the production of ATP during the conversion of ADP (adenosine 5’-diphosphate sodium salt, A2754, Sigma-Aldrich, USA) and CP (carbamoyl phosphate di-lithium salt, C5625, Sigma-Aldrich). For this, 2 mM ADP, 2 mM CP, and 100 nM YbcF (or lysate) were added to the reaction mixture containing 20 mM Tris-Cl (pH 6.0, 7.0, or 8.0), 30 mM MgCl_2_, and 0.02% BSA and incubated for 60 min at 28°C or 37°C.

To determine the concentration-dependent kinetics, CK activity was measured using 100 nM YbcF by varying the concentration of CP from 0 to 3 mM while holding ADP fixed at 2 mM, and vice versa. After 60 min at 28°C, the ATP concentration in the reaction mixture was measured with an ATP Determination Kit (A22066, Molecular Probes Inc., USA). The ATP standard (10 μl) or carbamate kinase assay sample (10 μl) was added to the Standard Reaction Solution (90 μl, 0.5 mM D-luciferin, 1.25 μg/ml firefly luciferase, 25 mM tricine buffer, pH 7.8, 5 mM MgSO_4_, 100 μM ethylenediaminetetraacetic acid, 1 mM dithiothreitol). Then they were incubated at 28°C for 15 min. The amount of ATP was analyzed with a Spectramax i3 bioluminescence reader (Molecular Devices, USA) at 560 nm.

The Michaelis-Menten constant (*K*_M_) and maximum velocity (*V*_max_) of ADP and CP were calculated using the Michaelis-Menten equation and Lineweaver-Burk equation, respectively.

### Statistical Analysis

Statistical analyses were performed using PASW Statistics 18 (SPSS, Inc.). Data were analyzed using unpaired two-tailed Student’s *t*-tests and one-way analysis of variance (ANOVA). Post hoc analyses were performed using Duncan’s multiple comparison test. Statistical significance was defined as *p* < 0.05.

## Results 

### The *ybcF* gene Is Part of the *fdrA* Operon

**Fig. 1:**
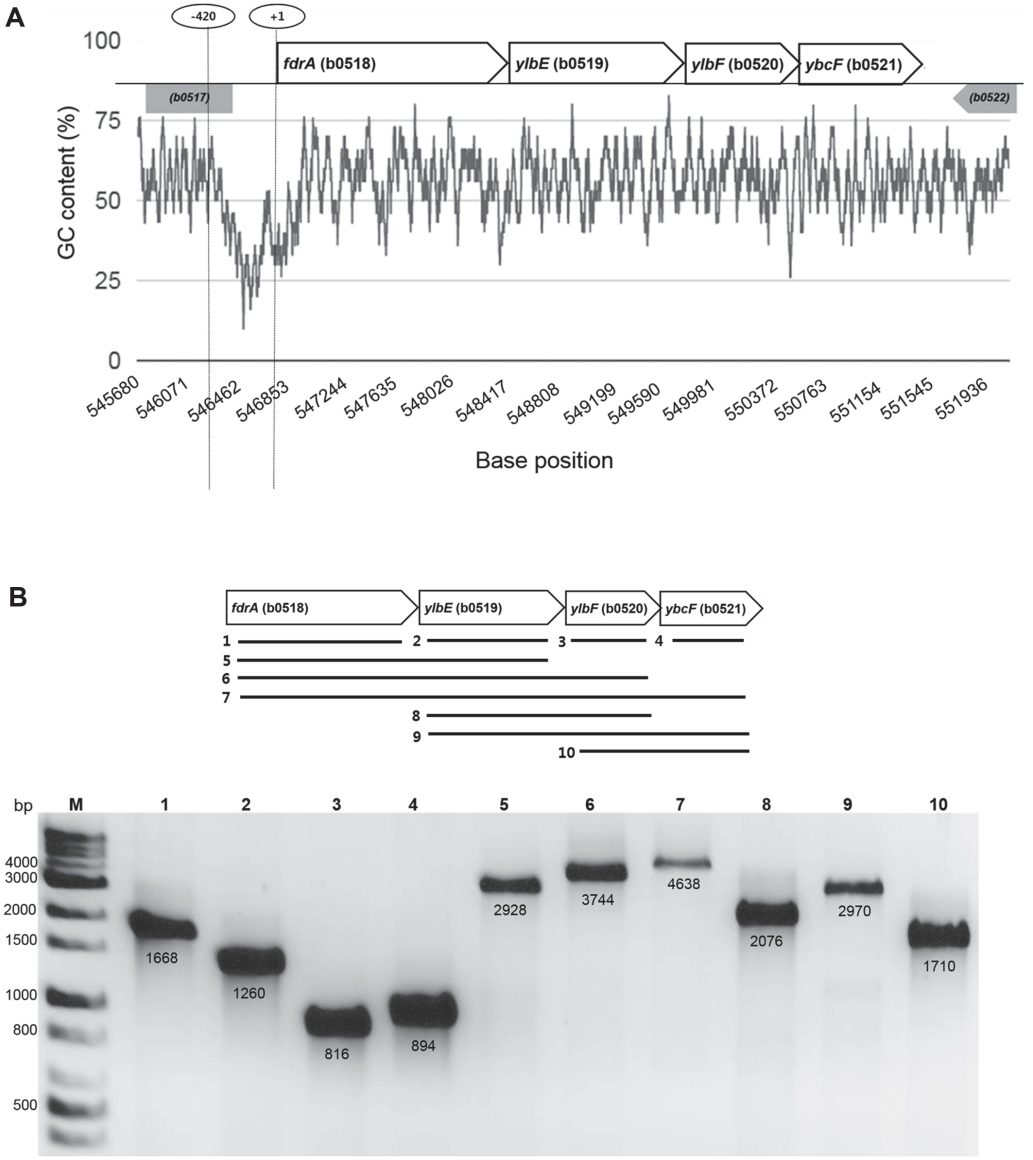
Validation of the *fdrA* operon. **A** Promoter region prediction (-420 ~ +1) by GC content (Biologics International Corp., https://www.biologicscorp.com/tools/GCContent/). **B** Searching for transcripts of *fdrA*, *ylbE*, *ylbF*, and *ybcF* in total mRNA of *E. coli* MG1655 by RT-PCR. Total mRNA isolated from *E. coli* was grown under anaerobic conditions for allantoin degradation was reverse-transcribed by RT-PCR into cDNA. The cDNA was amplified by PCR using primers located in the genes in various combinations. The calculated lengths of the products (bp) are given below the bands. M, 1-kb DNA ladder. Lane 1, *fdrA*; lane 2, *ylbE*; lane 3, *ylbF*; lane 4, *ybcF*; lane 5, *fdrA*-*ylbE*; lane 6, *fdrA*-*ylbE*-*ylbF*; lane 7, *fdrA*-*ylbE*-*ylbF*-*ybcF*; lane 8, *ylbE*-*ylbF*; lane 9, *ylbE*-*ylbF*-*ybcF*; lane 10, *ylbF*-*ybcF*.

The possible promoter regions for gene clusters in the *E. coli* genome around *ybcF* were investigated by calculating the G+C content percentage in GC Content Calculator (https://www.biologicscorp.com/tools/GCContent/). Low G+C content provides a relative low stacking energy between strands of double-stranded DNA, which enables it to open at the initiation of transcription. We found a low G+C content region ca. 400 bps in length in front of the *fdrA* gene, which is only the promoter region we expected. The region in front of *ybcF* shows slightly low G+C content, but it is very short (Fig. 1A). In addition, two bioinformatics tools using iPro54-PseKNC and iPro70-FMWin predicted binding motifs for δ54 and δ70 upstream 334 bps (score: 0.9121) and upstream 271 bps (score: 0.9978) from the *fdrA* gene, respectively, which were consistent with the G+C content prediction [21, 22].

We investigated and found that *ybcF* was co-transcribed with *fdrA*, *ylbE*, and *ylbF*, *i.e.*, they were transcribed to one polycistronic mRNA. Total mRNA was isolated from a wild-type strain of *E. coli* grown anaerobically on allantoin as the sole nitrogen source. The total mRNA was reverse-transcribed into cDNA that was then amplified with every conceivable combination of primer pairs that bind to the *fdrA*, *ylbE*, *ylbF*, and *ybcF* genes (Fig. 1B). The results showed that each of the four genes, *fdrA*, *ylbE*, *ylbF*, and *ybcF*, was well and comparably expressed under allantoin degradation conditions (lanes 1 to 4). Lanes 5 to 10 show that the total mRNA contained all possible combinations of these neighboring gene transcripts, including two-gene transcripts (*fdrA*-*ylbE*, *ylbE*-*ylbF*, *ylbF*-*ybcF*), three-gene transcripts (*fdrA*-*ylbE*-*ylbF*, *ylbE*-*ylbF*-*ybcF*), and the four-gene transcript (*fdrA*-*ylbE*-*ylbF*-*ybcF*), indicating that the *ybcF* gene belongs to the *fdrA* operon.

### Deletion of the *ybcF* Gene Does Not Affect Allantoin Degradation

**Fig. 2:**
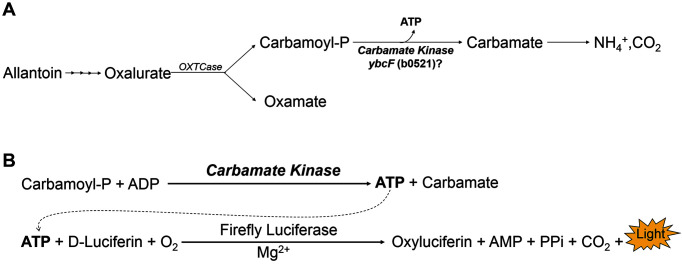
The anaerobic allantoin degradation pathway and determination of carbamate kinase activity. **A** The allantoin degradation pathway in which *ybcF* encodes the enzyme responsible for generating ATP. **B** The principle for determination of ATP production. Carbamoyl-P, carbamoyl phosphate, OXTCase, oxamic transcarbamylase.

Because *ybcF* is part of the *fdrA* operon, its expression will be controlled by a common promoter located upstream of *fdrA*. In a recent study, we analyzed the expression of the *fdrA* operon using *fdrA*-*lacZ* reporter gene fusion and found that its expression was dramatically induced by the presence of allantoin under anaerobic conditions [16]. Therefore, *ybcF* (as well as *fdrA*) was expected to play a role in the anaerobic degradation of allantoin (Fig. 2A). Moreover, Smith *et al*. used bioinformatic tools to consider the genome and metabolic context with sequence orthology and predicted that *ybcF* would encode a carbamate kinase [17]. Therefore, we also assumed that YbcF could be a catabolic carbamate kinase associated with anaerobic allantoin degradation. The proposed reaction would produce ATP from carbamoyl phosphate (CP): CP + ADP → carbamate + ATP. To verify that reaction, we cultivated the wild-type and Δ*ybcF* (LMB111) strains of *E. coli* anaerobically on allantoin with glycerol plus DMSO in nitrogen-deficient M9 medium for 48 h. However, no significant change in allantoin degradation due to *ybcF* deletion was observed (Table S1). Next, allantoin degradation by the Δ*ybcF* strain was observed using a concentrated cell suspension, and no noticeable changes were observed, similar to the results in growth culture (Table 1, Table S1). Allantoin is degraded into oxalurate and then divided into equivalent amounts of oxamate and CP (Fig. 2A). CP is a very unstable substance, whereas oxamate is a dead-end metabolite [23]. Therefore, we quantified oxamate and estimated that the same amount of CP was generated. It could be that the allantoin pathway was not interrupted in the Δ*ybcF* strain because of the rapid consumption of CP for arginine and pyrimidine biosynthesis under the tested nitrogen-deficient growth conditions. Therefore, we conducted the following direct carbamate kinase assay to identify the enzyme YbcF.

### *E. coli* YbcF Shows Activity as a Catabolic Carbamate Kinase (CK)

During anaerobic allantoin degradation, oxalurate is degraded into CP and oxamate by OXTCase (Fig. 2A). The phosphate group of CP is transferred to ADP by CK to produce ATP, leaving carbamate, which is then spontaneously mineralized to HCO_3_^-^ and NH_4_^+^ (Fig. 2A). The CK activity in this study was determined by quantifying the ATP produced, which we measured by coupling it to a recombinant firefly luciferase and its substrate D-luciferin (Fig. 2B) [24].

**Fig. 3:**
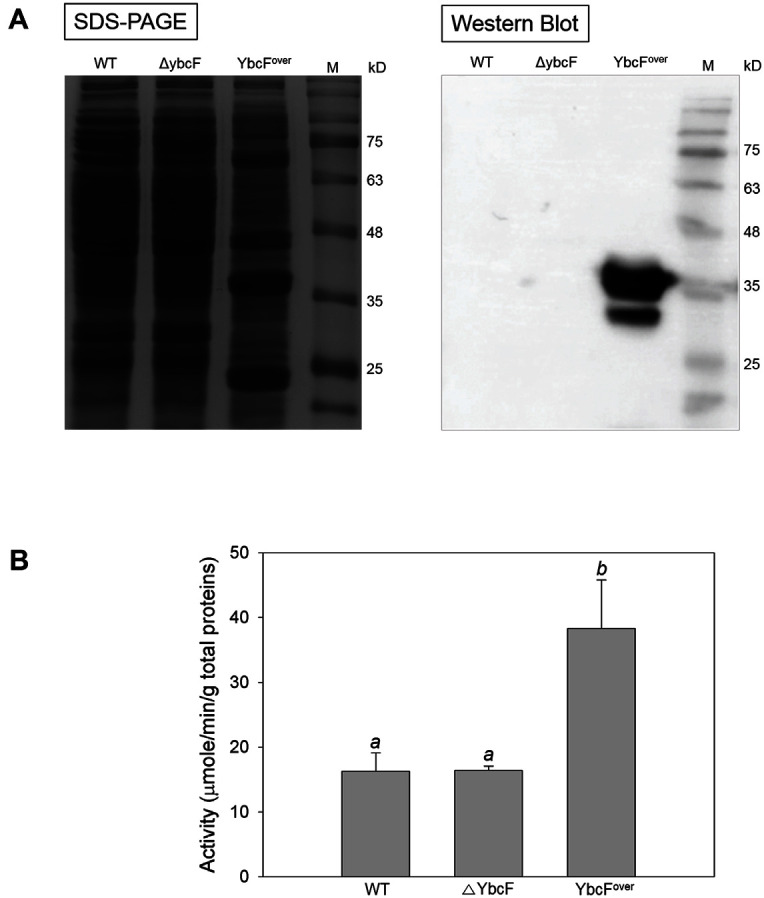
Evaluation of the carbamate kinase activity of YbcF using crude lysates of the wild-type (WT), *ybcF*deleted (Δ*ybcF*), and YbcF-overproducing (YbcF^over^) strains of *E. coli*. **A** SDS-PAGE gel (left) and Western blot membrane (right) show the expression of YbcF. Protein YbcF corresponds to 34.1 kDa, including six histidines. **B** The carbamate kinase activity of the lysate was calculated as moles of ATP produced per minute to total protein in g after reacting at 28°C for 60 min. The different superscripts indicate significant differences at p < 0.05. SPSS PAWS Statistics, one-way ANOVA, Duncan’s multiple comparisons. Values were determined from three replicates. Error bars indicate standard deviations.

First, the CK activity of YbcF was evaluated using crude cell lysate (Fig. 3). The cell lysate containing overproduced YbcF (Fig. 3A) clearly showed higher CK activity (38 μmole/min/g total protein) than the wild-type and Δ*ybcF* strains (about 16 μmole/min/g total protein) (Fig. 3B). The lack of difference in CK activity between wild-type and Δ*ybcF* strains could be due to the high background caused by the use of crude cell lysate, as reflected by the following two factors. This enzyme assay was unable to distinguish CK activity from acetate kinase and carbamoyl phosphate synthetase activity [25], and ATP was already present in the cell lysates. Therefore, their sum was measured as CK activity, and that value represented quite a high background. Thus, we found that YbcF had CK activity by using crude cell lysate from an YbcF-overexpressing strain.

### Carbamate Kinase Kinetics of YbcF

**Fig. 5:**
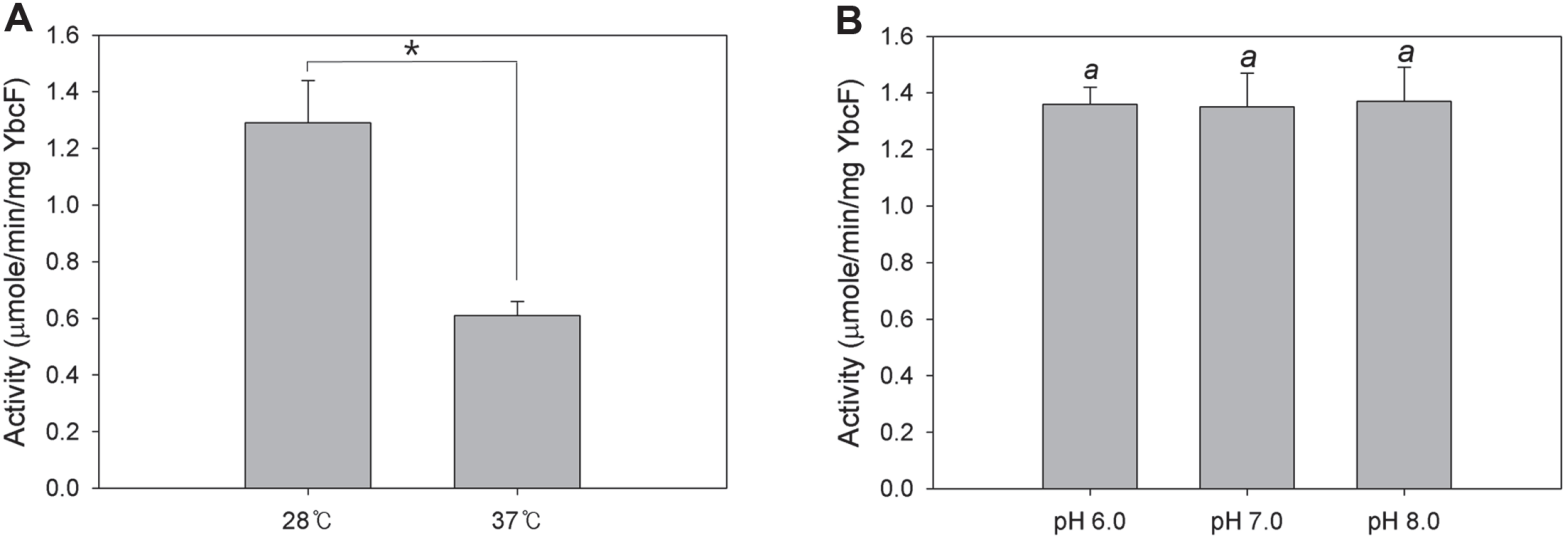
Carbamate kinase activity of purified YbcF depending on temperature A and pH B. **A** Asterisk ‘*’ indicates significant differences at *p* < 0.05. Unpaired two-tailed Student’s t-tests were performed to analyze the comparison. **B** Identical letters in respective pH conditions indicate no significant differences at *p* < 0.05. SPSS PAWS Statistics, one-way ANOVA, Duncan’s multiple comparisons. Values were determined from three replicates. Error bars indicate standard deviations.

The optimal temperature and pH value for the CK enzyme assay were determined with purified YbcF. The overproduction and purification of YbcF were confirmed by SDS-PAGE and Western blotting (Fig. 4), and 100 nM YbcF was used for each assay. The temperature was tested at 28°C and 37°C, the former being the recommended temperature for ATP quantification (A22066), and the latter the optimum temperature for the carbamate kinase reaction (E.C.2.7.2.2). Because CK activity was twice as high at 28°C (1.29 μmole/min/mg YbcF) than at 37°C (0.61 μmol/min/mg YbcF), the temperature selected was 28°C (Fig. 5A). The chosen pH value was pH 8 (1.37 μmole/min/mg YbcF). Although there was no significant difference at pH 6, 7, and 8 (Fig. 5B), the luminescence-based CK assay previously was performed at pH 8 [24].

**Fig. 6:**
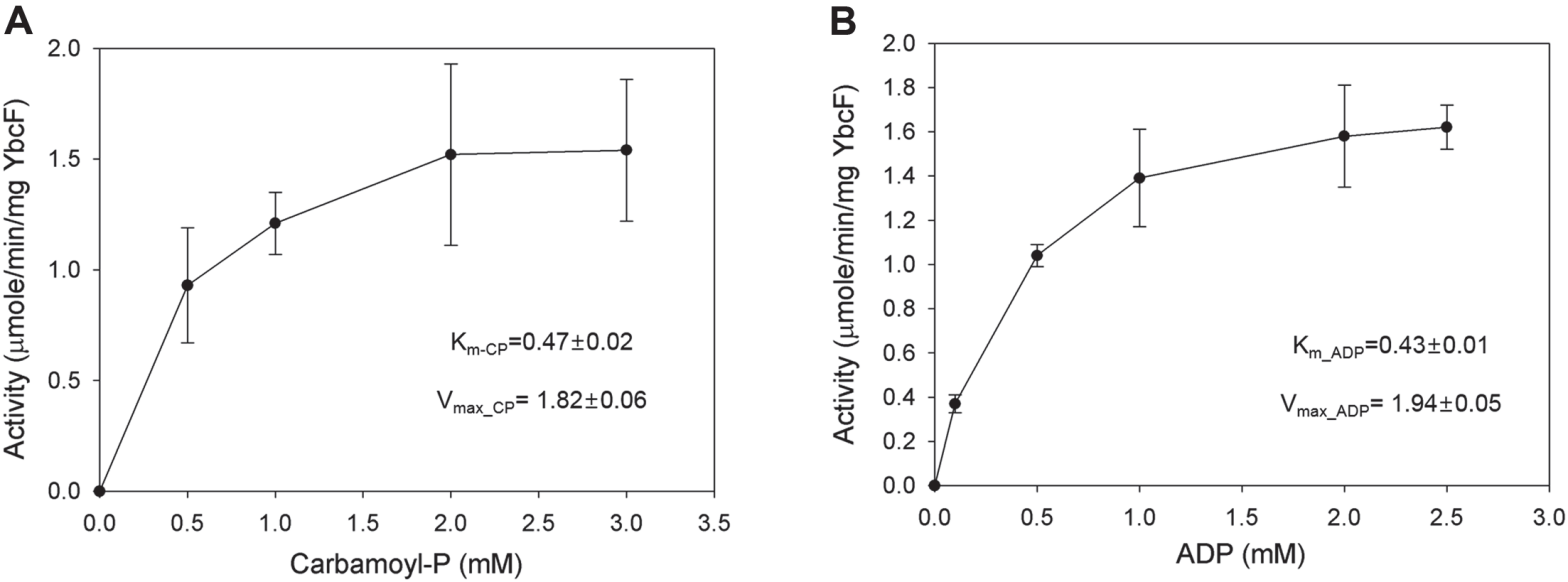
Carbamate kinase activity of purified YbcF depending on the concentrations of carbamoyl phosphate A and ADP B. The kinetic parameters *V*_max_ (maximum velocity) and *K*_M_ (Michaelis-Menten constant) for carbamate kinase were calculated from the Lineweaver-Burk equation and Michaelis-Menten equation, respectively. Values were determined from three replicates. Error bars indicate standard deviations.

The CK kinetics of YbcF were investigated at 28°C and pH 8 for 60 min, and we calculated the amount of ATP produced as U/mg YbcF protein. CK has two substrates in the forward direction for allantoin degradation: CP and ADP. Thus, CK activity was measured depending on the concentration of those two substrates. Keeping ADP fixed at 2 mM, activity was measured as we changed the concentration of CP from 0–3 mM, and vice versa (Fig. 6). When CP was varied, *V*_max_CP_ was 1.82 μmol/min/mg YbcF, and *K*_M_CP_ was 0.47 mM. The values were similar when ADP was varied: *V*_max_ADP_ was 1.94 μmol/min/mg YbcF, and *K*_M_ADP_ was 0.43 mM (Fig. 6).

## Discussion

We found in this study that catabolic CK is encoded by the *ybcF* gene belonging to an operon (b0518–0521) downstream of the allantoin cluster in the *E. coli* genome. That operon begins with the *fdrA* gene encoding a potential OXTCase, a catabolic transcarbamoylase [16]. This is somehow similar to that in *L. brevis*, where the *arcC* encoding the catabolic CKs is located together with the arcB for the catabolic transcarbamoylase OTCase in the ADI operon [12]. However, the catabolic CK of *E. coli* (*ybcF*) should be distinguished from *arcC* of *L. brevis* in that it is on the allantoin degradation pathway, not the arginine deiminase pathway.

The *K*_M_ value of YbcF (0.47 mM) for CP is relatively low compared with those of catabolic CKs determined in other bacteria: *S. pyogenes* (0.65 mM), *E. faecalis* (1.40 mM), *P. aeruginosa* (5 mM), and *L. buchneri* (1.1 mM, 1.53 mM) (Table 2) [26-29]. On the other hand, in CKs of two parasitic/pathogenic protozoa, the *K*_M_ values for CP are very low, *G. intestinalis* (0.085 or 0.34 mM) and *T. vaginalis* (0.13 mM) (Table 2), which is thought to indicate that they evolved to easily use the energy-rich CP (ΔG^0′^ = -39 kJ/mol) produced by the host as an energy source [30-32]. In addition, the *K*_M_ value of YbcF in *E. coli* for ADP (0.43 mM) is similar to that for CP (0.47 mM) (Table 2), showing that YbcF has similar affinity for the two substrates, so it seems easy to perform catabolic CK. Overall, the *K*_M_ values indicate that YbcF, as a catabolic CK, has a higher affinity for the substrates CP and ADP than those of other nonpathogenic bacteria.

**Fig. 7:**
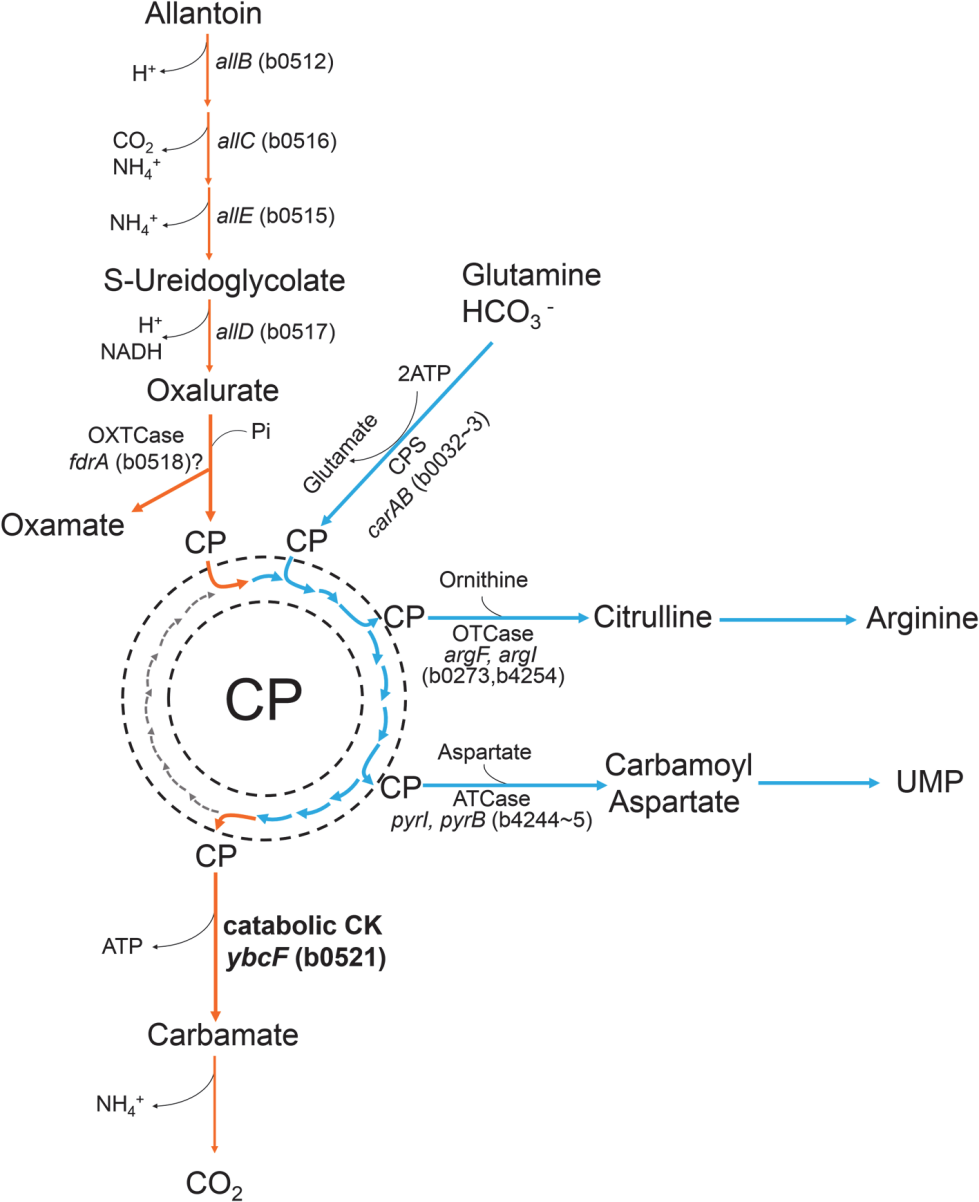
Schematic diagram of the intersectional fate of carbamoyl phosphate. In the allantoin degradation pathway, *ybcF* encodes the enzyme responsible for generating ATP by transferring a phosphate group from carbamoyl phosphate to ADP. The carbamoyl phosphate that originated from glutamine via carbamoyl phosphate synthetase is used in the biosynthesis of arginine and uridine monophosphate (UMP). OXTCase, oxamic transcarbamylase; CP, carbamoyl phosphate; CPS, carbamoyl phosphate synthetase; OTCase, anabolic ornithine transcarbamoylase; ATCase, aspartate transcarbamoylase; catabolic CK, catabolic carbamate kinase; orange path, catabolism; blue path, anabolism.

*E. coli* can use allantoin, the main product of purine degradation, as a sole nitrogen source, but only under anaerobic conditions [33]. The conversion steps from allantoin to oxalurate are ring opening, deamination, and oxidation, and they are catalyzed by enzymes encoded by *allB* (b0512), *allC* (b0516), *allE* (b0515), and *allD* (b0517) (Fig. 7, upper vertical route). Subsequently, oxalurate is converted to oxamate and CP by potential OXTCase (probably *fdrA*, b0518), and CP is dephosphorylated by CK (b0521) to carbamate, which spontaneously degrades (Fig. 7, lower vertical route). Alternatively, ureidoglycolate is divided into urea and glyoxylate by ureidoglycolate lyase (*allA*, b0505), and the latter is then oxidized to oxalate [16, 33]. We predicted that the *ybcF* deletion strain (Δ*ybcF*) would not decompose CP, and the path would be bypassed from ureidoglycolate to glyoxylate, resulting in an increase in oxalate and decrease in oxamate (and CP). Although changes that matched those predictions did occur, the decrease in oxamate (and CP) was very slight (Table 1, Table S1), indicating that CP continues to be used in Δ*ybcF* mutant. The CP used in biosynthesis is produced by CPS in *E. coli*, with the amino group coming from glutamine, and the reaction requires 2 ATP (Fig. 7). Furthermore, the culture medium used in this study contained no nitrogen source other than allantoin. Therefore, if CP is available in the cell, it would be beneficial to use it first. Compared with CK YbcF (*K*_M_ 0.47 mM), both of the OTCase isoenzymes, ArgF (*K*_M_ 0.36 mM) and ArgI (*K*_M_ 0.05 mM), exhibit higher affinity for CP in arginine biosynthesis (Fig. 7, Table 3). Also, ATCase (*K*_M_ 0.20 mM) shows a higher affinity for CP in pyrimidine biosynthesis (Fig. 7, Table 3). Therefore, the CP in the cell is preferentially used for de novo synthesis of arginine or pyrimidine, and the remaining CP is used for ATP generation via the catabolic carbamate kinase because energy sources are rare in anaerobic environments.

## Supplemental Materials

Supplementary data for this paper are available on-line only at http://jmb.or.kr.

## Figures and Tables

**Fig. 4:**
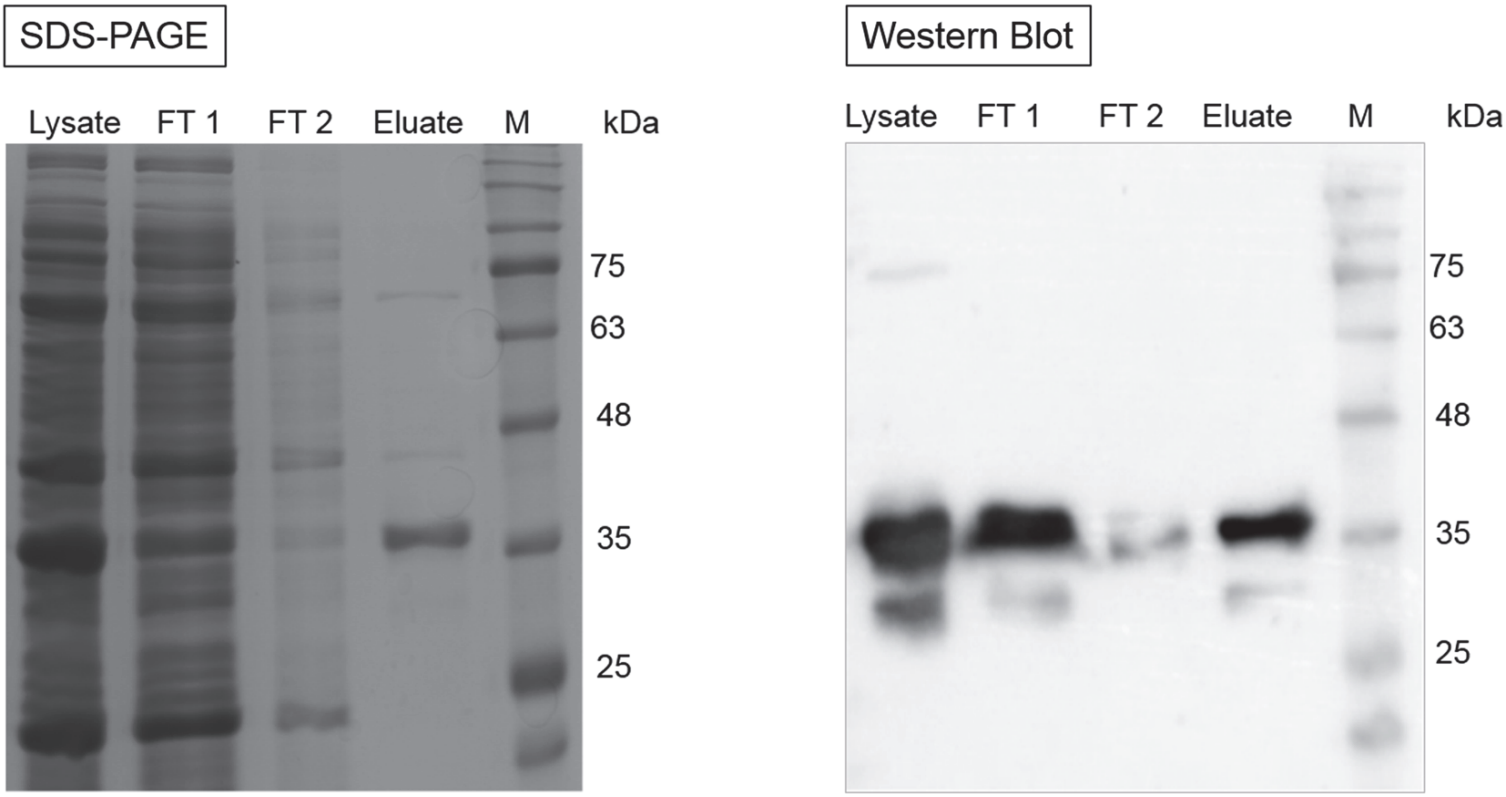
4. Purification of YbcF. Left panel, SDS-PAGE; right panel, Western blot; Lysate, lysate of *E. coli* MG1655pCA24N::ybcF; FT 1, flow through fraction; FT 2, washing fraction; Eluate: elution fraction of YbcF; M, protein ladder.

**Table 1:** Anaerobic allantoin degradation in a cell suspension of wild-type and Δ*ybcF* strains of *E. coli*.

		WT			Δ*ybcF*	

Time (min)	Allantoin	Oxamate	Oxalate	Allantoin	Oxamate	Oxalate
0	13.4 ± 0.9^*a*,*b*^	0	0	13.7 ± 1.0	0	0
10	8.9 ± 0.9	7.1 ± 0.9	0.8 ± 0.1	8.9 ± 1.3	5.8 ± 0.9	1.2 ± 0.1
20	4.2 ± 0.9	14.2 ± 1.4	1.2 ± 0.5	4.2 ± 1.5	12.6 ± 1.4	2.0 ± 0.3
30	0.3 ± 0.5	20.6 ± 2.6	1.6 ± 0.5	0.4 ± 0.6	19.0 ± 4.4	2.5 ± 0.5
60	0	21.3 ± 4.2	1.6 ± 0.6	0	19.8 ± 5.6	2.6 ± 0.5
Rate for consumption or formation at 20 min (mmol/gCDW/h)	10.5 ± 0.4	16.0 ± 0.8	1.3 ± 0.6	10.9 ± 0.6	14.5 ± 2.0	2.3 ± 0.3

The bacterial strains were anaerobically grown on glycerol (50 mM), DMSO (50 mM), and allantoin (20 mM) in nitrogendeficient M9 medium and resuspended in nitrogen/carbon-deficient MOPS buffer to an OD_600_ of 20. The reaction was started by adding allantoin (20 mM), glycerol (20 mM), and DMSO (20 mM).

WT, wild-type; Δ*ybcF*, *ybcF* deletion mutant; gCDW, g cell dry weight

The OD_600_ of the cell suspension maintained; initial pH value of 7.0 increased to 7.4 at the end of incubation of both WT and Δ*ybcF*.

^a^Unpaired two-tailed student’s *t*-tests were performed to analyze WT and Δ*ybcF*.

^b^Values represent avg ± SD for 3 replicates.

**Table 2:** The *K*_M_ values for carbamoyl phosphate (CP) and ADP of known catabolic carbamate kinases.

Organisms	Genes	*K*_M_ for CP (mM)	*K*_M_ for ADP (mM)	References
*Escherichia coli*	*ybcF*(*arcC*)	0.47	0.43	This study
*Streptococcus pyogenes*	*arcC*	0.65	0.72	[26]
*Enterococcus faecalis*	*arcC1*	1.40	0.04	[27]
*Pseudomonas aeruginosa*	*arcC*	5	0.3	[28]
*Lactobacillus buchneri*	*arcC*	1.53	0.71	[29]
		1.1	0.57	[34]
*Giardia intestinalis^a^*	*CBK*	0.34	N.A.	[31]
		0.085	0.07	[30]
*Trichomonas vaginalis^a^*	*CBK*	0.13	N.A.	[32]

N.A. not available.

^a^Parasitic/pathogenic eukaryotic microorganism.

**Table 3:** The *K*_M_ values of *E. coli* enzymes using carbamoyl phosphate as substrate.

Enzyme	*K*_M_ for CP (mM)	Genes	References
catabolic Carbamate Kinase (CK)	0.47	*ybcF*	This study
Aspartate Transcarbamoylase (ATCase)	0.20	*pyrB*	[35]
Ornithine Transcarbamoylase (OTCase)	0.05	*argI* (OTCase-1)	[36]
	0.36	*argF* (OTCase-2)	[37]
