# Supplementary material for: The ybcF Gene of Escherichia coli Encodes a Local Orphan Enzyme, Catabolic Carbamate Kinase
Source: J Microbiol Biotechnol. 2022 Nov 7;32(12):1527–36. doi: 10.4014/jmb.2210.10037 (PMC9843812; doi:10.4014/jmb.2210.10037)
Supplement: Supplementary file 1 [file jmb-32-12-1527-supple.pdf]

J. Microbiol. Biotechnol.
https://doi.org/10.4014/jmb.2210.10037

Supplementary Table

The *ybcF* gene of *Escherichia coli* encodes a local orphan enzyme, catabolic carbamate kinase

Nam Yeun Kim and Ok Bin Kim

Department of Life Science, Division of EcoScience, Ewha Womans University, Seoul 03760, Republic of Korea

Corresponding author:

[#] Ok Bin Kim

Email: kimokbin@ewha.ac.kr,

Table S1. The metabolic profiles of wild-type and *ΔybcF* (LMB111) strains of *E. coli* during anaerobic growth on glycerol (50 mM), DMSO (50 mM), and allantoin (20 mM) in nitrogen-deficient M9 medium for 48 h.

		WT	*ΔybcF*
Consumption (mM)	Glycerol	21.6 ± 1.1[a, b]	19.4 ± 1.3
	Allantoin	21.0 ± 0.8	21.0 ± 0.9
Production (mM)	Oxamate	15.4 ± 2.5	13.1 ± 0.8
	Oxalate	3.8 ± 0.6	4.4 ± 0.1
	Succinate	4.2 ± 0.4	4.2 ± 0.6
	Formate	23.7 ± 2.5	21.3 ± 1.2
	Acetate	12.2 ± 1.6	12.3 ± 0.1
	Ethanol	13.2 ± 0.5	14.0 ± 0.9
Growth	OD_{600}	0.7 ± 0.1	0.7 ± 0
	pH	6.6 ± 0	6.7 ± 0.1

WT, wild-type; *ΔybcF*, *ybcF* deletion mutant

[a]Unpaired two-tailed Student's t-tests were performed to analyze WT and *ΔybcF*.

[b]Values represent avg ± SD of three replicates.
